# Assessing the environmental impact of clear aligner therapy: a scoping review

**DOI:** 10.3389/fdmed.2026.1849048

**Published:** 2026-08-24

**Authors:** Sapthami Pai, Dhruv Ahuja, Ashith MV, Haneesh Kiran PT, Amoli Singh

**Affiliations:** 1 Department of Orthodontics and Dentofacial Orthopaedics, Manipal College of Dental Sciences Mangalore, Manipal Academy of Higher Education, Manipal, Karnataka, India; 2 Department of Orthodontics and Dentofacial Orthopedics, Manav Rachna Dental College, Manav Rachna International Institute of Research and Studies, Haryana, India; 3 Department of Orthodontics and Dentofacial Orthopaedics, Yenepoya Dental College (Yenepoya Deemed to be University), Mangalore, Karnataka, India

**Keywords:** aligners, microplastics, orthodontics, sdgs, sustainability

## Abstract

**Objective:**

To evaluate the environmental impact and sustainability-related practices of clear aligner therapy (CAT), focusing on environmental impact, economic implications, waste management, recycling practices, manufacturing processes, and aligner materials. It further explores the alignment of clear aligner practices with Sustainable Development Goals (SDG's) 3,9,12 and 13.

**Methodology:**

A scoping review was conducted following the Preferred Reporting Items for Systematic Reviews and Meta-Analyses Extension for Scoping Reviews (PRISMA-ScR) guidelines. The review was guided by the Population–Concept–Context (PCC) framework. A comprehensive literature search was performed across five electronic databases (PubMed Central, Google Scholar, Scopus, Web of Science, and EBSCOhost) for studies published between January 2014 and December 2024. Studies evaluating the environmental sustainability of clear aligner therapy, including materials, manufacturing processes, waste management, recycling practices, and carbon emissions, were included.

**Results:**

Thirteen studies met the inclusion criteria. The evidence indicates that clear aligner therapy contributes to plastic waste, microplastic generation, and greenhouse gas emissions throughout its life cycle. The included studies reported biodegradable materials, direct three-dimensional (3D) printing technologies (SDG-9), recycling initiatives (SDG 12), and circular economy approaches as potential strategies to reduce environmental impacts (SDG 3 & 13). However, the implementation of these strategies remains constrained by limited recycling infrastructure, economic considerations, and insufficient high-quality evidence.

**Conclusion:**

Available evidence suggests that innovations in material selection, circular economy approaches, optimized manufacturing processes, and enhanced recycling programs may reduce the environmental burden associated with clear aligner therapy. Nevertheless, further high-quality environmental assessment studies are required to support the development of more sustainable orthodontic practices aligned with SDGs 3, 9, 12, and 13.

## Introduction

The growing demand for aesthetic orthodontic treatment has driven the widespread adoption of clear aligner therapy (CAT) across different age groups. Clear aligners have become an increasingly popular alternative to conventional fixed orthodontic appliances because of their improved aesthetics, patient comfort, ease of oral hygiene maintenance, and enhanced treatment acceptance. During clinical use, aligners are exposed to fluctuations in temperature, saliva, oral microorganisms, and mechanical forces such as mastication and bruxism, all of which may influence their physical and mechanical properties. Clear aligner therapy is a comfortable, discreet alternative to braces, popular for its ease of cleaning, reduced tissue irritation, and enhanced compliance (1). Prolonged oral exposure subjects them to temperature changes, bacteria, and mechanical wear from habits like bruxism (2).

The material composition of clear aligners determines their mechanical performance, transparency, durability, and clinical effectiveness. The most commonly used polymers include polyethylene terephthalate glycol (PET-G), polypropylene (PP), polycarbonate (PC), thermoplastic polyurethane (TPU), and ethylene-vinyl acetate (EVA), each possessing distinct physical and mechanical characteristics that influence clinical performance (3). As these petroleum-based polymers undergo degradation during use and disposal, they may contribute to the release of microplastics into the environment, highlighting the need to evaluate their environmental implications alongside their clinical performance (4). Bisphenol-A (BPA), found in epoxy resins and polycarbonates used in some aligners, may leach due to intraoral degradation caused by temperature, pH, or wear. BPA is linked to estrogenic effects, toxicity, and cell proliferation (5, 6). While the Environmental Protection Agency (EPA) sets a safe intake limit of 50 µg/kg, adverse effects have been reported even below this threshold (6). Some studies show no harmful effects of BPA release from aligners, while others report negative outcomes (2). Ravuari et al. (7), conducted reviews on the biocompatibility, safety, and potential BPA-related risks in aligners, examining aspects like cytotoxicity and estrogenic effects.

The increasing use of plastic-based materials in clear aligner therapy has raised concerns regarding their environmental impact throughout the product life cycle. The manufacture, transportation, clinical use, and disposal of aligners contribute to plastic waste generation, greenhouse gas emissions, and the release of microplastics into terrestrial and aquatic ecosystems. These environmental challenges have prompted increasing attention toward sustainable material development, improved waste management, and circular economy strategies within orthodontics (8). Consequently, evaluating the environmental impact of clear aligners is directly relevant to several United Nations Sustainable Development Goals (SDGs), particularly SDG 3 (Good Health and Well-being), SDG 9 (Industry, Innovation and Infrastructure), SDG 12 (Responsible Consumption and Production), and SDG 13 (Climate Action) (9, 10). Briefly, SDG 3 (Good Health and Well-being) seeks to ensure healthy lives and promote well-being for all ages, which is relevant given the potential health implications of microplastic and Bisphenol-A exposure arising from aligner materials. SDG 9 (Industry, Innovation and Infrastructure) promotes resilient infrastructure, sustainable industrialisation, and innovation, reflected in emerging approaches such as direct three-dimensional printing of aligners. SDG 12 (Responsible Consumption and Production) calls for sustainable consumption and production patterns, including efficient use of resources, waste reduction, and environmentally sound management of materials, which underpins recycling and circular economy strategies for aligners. SDG 13 (Climate Action) calls for urgent action to combat climate change and its impacts, which is pertinent to the greenhouse gas emissions generated during the manufacture, transport, and disposal of aligners.

As the demand for clear aligners grows, so does the need for awareness of their environmental impact. Aligners, being single-use products, contribute significantly to plastic waste, with each set being replaced based on the clinician's preferences or the manufacturer's recommendations. The materials used in their production such as PET-G, TPU, and PP are durable but not easily recyclable. This leads to substantial waste, and as these materials degrade, they contribute to microplastic pollution, impacting both ecosystems and human health. Moreover, aligner production and disposal, including processes like incineration or landfilling, release greenhouse gases, adding to the environmental burden (11). However, several strategies can mitigate these impacts. For example, intraoral scanners have become an essential tool in orthodontics, reducing the need for physical impressions and minimising associated plastic waste (12).

Economic and material considerations used to manufacture aligners, such as PET-G, PC, and TPU, are chosen for their durability and biocompatibility, but these materials also have a direct impact on production costs. More durable materials can increase manufacturing expenses, raising the overall treatment cost for patients. However, advancements in manufacturing technologies and mass production are helping to bring these costs down. Additionally, there is growing interest in biodegradable materials, though they tend to be more expensive initially. These economic factors must be balanced against the environmental benefits, creating a complex dynamic for orthodontic manufacturers and practitioners (13, 14).

Recycling and sustainability practices for clear aligners presents a challenge due to their medical-grade plastic composition. Current recycling initiatives, allow for the downcycling of aligners into other plastic products, but they do not facilitate a closed-loop system for producing new aligners. The waste produced during aligner manufacturing, including flexible plastic packaging and cases, is repurposed in various ways such as energy recovery or turned into secondary products like floor tiles. There is, however, an increasing focus on 3D printing aligners directly from resin, reducing scrap and waste and offering a more sustainable manufacturing process (13, 14).

Therefore, this scoping review aimed to map the available evidence regarding the environmental impact of clear aligner therapy, including aligner materials, manufacturing processes, waste management, recycling practices, carbon emissions, and their relationship with Sustainable Development Goals (SDGs) 3, 9, 12, and 13. The review was guided by the following research question: “What evidence is available regarding the environmental impact and sustainability-related practices associated with clear aligner therapy?”

## Materials and methods

This scoping review was conducted in accordance with the Preferred Reporting Items for Systematic Reviews and Meta-Analyses Extension for Scoping Reviews (PRISMA-ScR) guidelines (15). The review followed the Population–Concept–Context (PCC) framework recommended for scoping reviews. The Population comprised patients undergoing clear aligner therapy; the Concept focused on the environmental sustainability of clear aligners, including materials, manufacturing, waste management, recycling, and carbon emissions; and the Context included orthodontic practice and the manufacture, use, and disposal of clear aligners. Two independent reviewers screened the studies at the title, abstract, and full-text stages according to predefined eligibility criteria.

### Search strategy

A comprehensive literature search was conducted across five electronic databases: PubMed Central, Scopus, Web of Science, Google Scholar, and EBSCOhost. The search included studies published between January 2014 and December 2024. Searches were performed using combinations of Medical Subject Headings (MeSH), keywords, and Boolean operators related to clear aligners, environmental sustainability, waste management, recycling, manufacturing processes, plastic pollution, carbon emissions, and orthodontics.

The principal search strategy included the following terms: (“clear aligners” OR “orthodontic aligners”) AND (“environmental impact” OR sustainability OR recycling OR “waste management” OR “plastic pollution” OR “carbon emissions”) AND (orthodontics OR dentistry). The search strategy was adapted for each database according to its indexing system and search syntax. In addition, the reference lists of the included articles were manually screened to identify further relevant studies.

All retrieved records were exported to Zotero (version 7), where duplicate records were identified and removed automatically, followed by manual verification. Two reviewers independently screened titles and abstracts, after which potentially eligible full-text articles were assessed against the predefined eligibility criteria. Any disagreements regarding study selection were resolved through discussion until consensus was achieved (16).

### Eligibility criteria

Studies were eligible if they were published as full-text articles in English between January 2014 and December 2024 and investigated the environmental sustainability of clear aligner therapy. Eligible studies evaluated one or more aspects of aligner materials, manufacturing processes, waste management, recycling practices, carbon emissions, life-cycle considerations, or other environmental impacts associated with clear aligner therapy.

Studies were excluded if they focused exclusively on clinical treatment outcomes, biomechanics, patient satisfaction, or material properties without addressing environmental sustainability. Conference abstracts, editorials, letters to the editor, duplicate publications, and studies lacking sufficient information relevant to the review objectives were also excluded. (Table 1)

**Table 1:** Inclusion and exclusion criteria used for study selection.

Inclusion criteria	Exclusion criteria
Full-text articles	Conference abstracts
English-language studies	Non-English publications
Published between January 2014 and December 2024	Duplicate publications
Studies on clear aligner therapy	Studies on fixed orthodontic appliances only
Environmental sustainability outcomes	Clinical effectiveness or biomechanics only
Materials, manufacturing, waste management, recycling, carbon emissions	Studies unrelated to environmental sustainability

### Data extraction and synthesis

Data extraction was independently performed by two reviewers (AMV & HK) using a standardized data extraction form. Information collected included author, year of publication, study design, sustainability focus, materials evaluated, environmental outcomes, manufacturing processes, recycling strategies, and principal findings. Disagreements were resolved through discussion until consensus was achieved.

Extracted data were analyzed using descriptive thematic synthesis. Studies were grouped according to the principal sustainability themes identified across the literature, including aligner materials, manufacturing processes, waste management and recycling, carbon emissions, and environmental impacts. Findings within each theme were compared to identify common patterns, differences, and gaps in the existing evidence.

## Results

**Figure 1:**
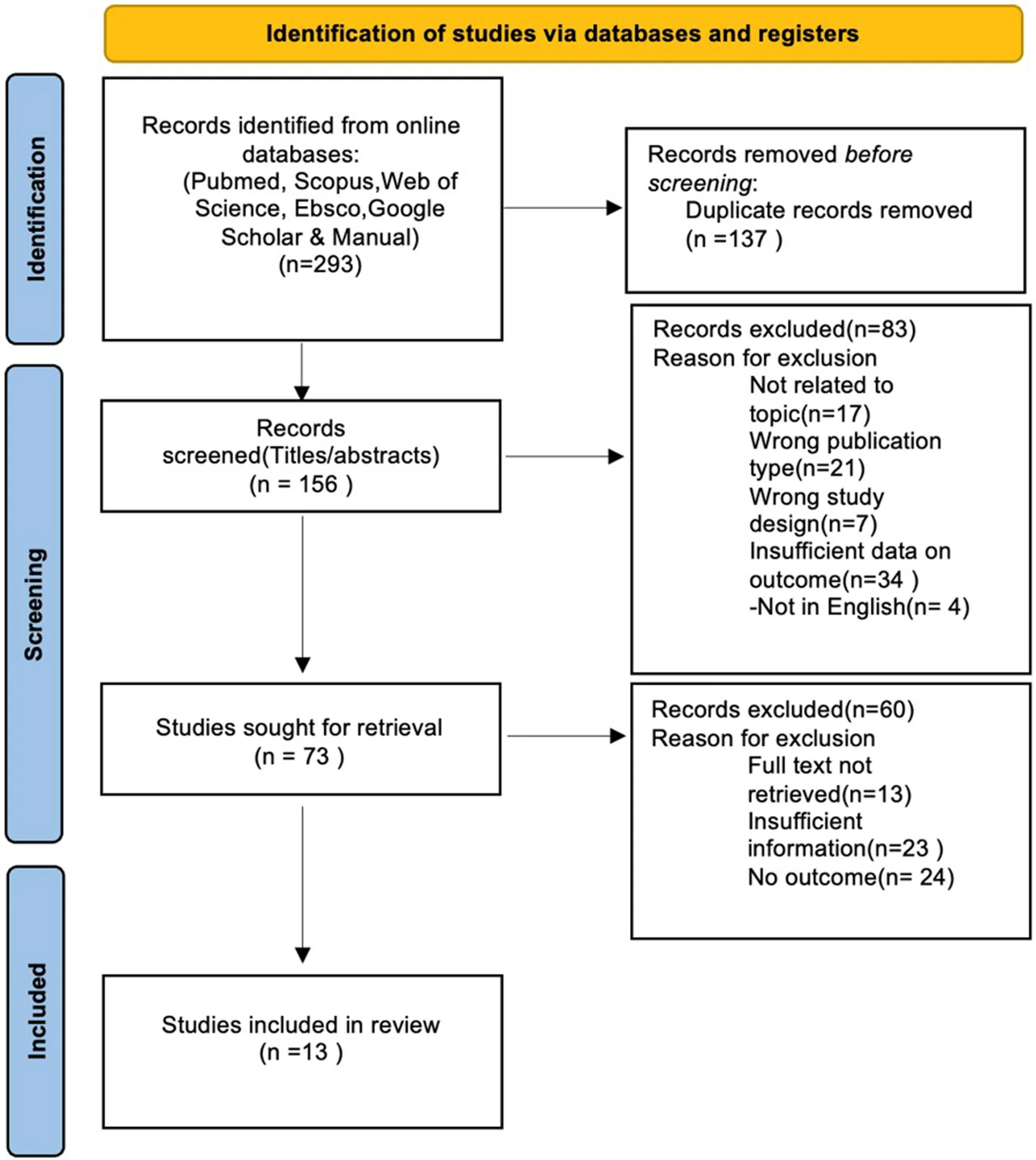
PRISMA flowchart for included studies.

The selection process followed the PRISMA flow diagram to depict the steps of article inclusion. After removing duplicates and irrelevant abstracts from the databases, 73 studies were screened by two independent reviewers. Disagreements regarding inclusion were resolved through discussion to reach a consensus. Ultimately, 13 studies were identified as relevant and selected for further analysis based on their alignment with the review's objectives. (Figure 1)

### Study characteristics

The 13 included studies were published between 2014 and 2024 and primarily consisted of narrative reviews, scoping reviews, environmental assessments, and laboratory-based investigations. The studies addressed a range of sustainability-related themes, including aligner materials, manufacturing technologies, waste management, recycling practices, carbon emissions, and environmental impacts associated with clear aligner therapy. A summary of the characteristics and principal findings of the included studies is presented in Table 2.

**Table 2:** Literature review on sustainability in clear aligner therapy.

S. No.	Authors	Sustainability type	Sustainability aspect	Findings
1.	Macrì et al., (2024) (17)	Environmental	Aligner materials Manufacturing process	This article examined the environmental impact of CAT in orthodontics and highlights strategies for reducing its ecological footprint. It proposes applying the 4Rs (reduce, reuse, recycle, rethink) to minimize resource use, incorporate recycled materials, and promote proper disposal and recycling of aligners. Emerging technologies and materials, along with initiatives for aligner recycling, offer solutions to reduce plastic waste and support a circular economy, while maintaining high-quality patient care.
2.	Ong et al., (2024) (18)	Environmental and economical	Waste management Recycling Aligner cost	This article highlights the challenges of dental plastic waste, advocating for sustainable alternatives like Polyhydroxyalkanoates (PHA) and Polylactic Acid (PLA), which are more costly than traditional plastics. Despite disposal and recyclability issues, compostable materials like PHAs show promise in reducing environmental impact, though cost and material diversity need further development.
3.	Panayi et al., (2024) (13)	Environmental	Microplastics Health hazards	Plastic aligners offer a discreet orthodontic solution, but concerns arise from the microplastics they release, which can have adverse effects on human health, including inflammation, oxidative stress, and metabolic disorders. To address these risks, a multidisciplinary approach is necessary, combining scientific research, regulatory oversight, and patient education to better understand the health and environmental impacts of microplastic exposure. Advancing research and promoting responsible practices will help mitigate potential risks associated with aligners, ensuring patient safety and reducing environmental harm
4.	Veseli et al., (2024) (11)	Environmental	Carbon emission	The nonbiodegradable nature of aligner plastics poses significant environmental concerns, including long decomposition times and harmful emissions from incineration. Solutions like hybrid aligner treatment to reduce aligner usage, recycling programs, and patient awareness can help address these challenges. Collaboration among dental professionals, manufacturers, and patients is essential to develop sustainable orthodontic practices while maintaining treatment efficacy.
5.	Akhtar et al., (2024) (19)	Environmental	Microplastics Health hazards	Investigated microplastic levels in dental units, revealing higher microplastic exposure in teaching hospitals compared to clinical settings, with seasonal variations. The analysis found polyethylene terephthalate to be the most common polymer, and the inhalation risk for dental professionals, particularly females, was identified as a concern. The Polymer Hazard Index (PHI) suggested a minor to medium health risk, highlighting the urgent need for sustainable practices in dental care, such as non-plastic protective equipment and better ventilation, to minimize microplastic exposure.
6.	Peter et al., (2024) (20)	Environmental	Aligner materials Manufacturing process	This study analyzed the smoke produced from incinerating two common aligner materials, PET-G and polyurethane. It identified hazardous compounds such as benzene (a carcinogen) in PET-G and tetrahydrofuran (a suspected carcinogen) in polyurethane, with several other irritants present. The study suggests mitigating strategies, including responsible disposal, 3D printing, and the development of biodegradable materials, as well as the establishment of aligner collection points to promote safe disposal practices.
7.	Hackley et al., (2024) (21)	Environmental and economical	Waste management Aligner cost Carbon emission	Environmental sustainability should be a key focus for dentists, as they play an essential role in minimizing the environmental impact of the oral healthcare industry. By adopting sustainable practices such as reducing travel emissions, managing resources responsibly, and prioritizing prevention, dental professionals can contribute to a healthier planet and improve the overall quality of care, fostering a more sustainable future for all.
8.	Gandhi et al., (2023) (22)	Environmental	Waste management Recycling	The growing demand for clear aligners has raised concerns about their environmental impact, as these non-biodegradable polyethylene terephthalate (PET) trays can persist in landfills for up to 100 years, with over 25 million aligners discarded annually. Improper disposal, often as general waste, risks infection spread and harmful emissions from incineration. Orthodontists play a key role in educating patients on safe disposal practices, such as returning used aligners for sterilization and recycling. Mechanical recycling, which converts discarded aligners into granules for other uses, offers a sustainable solution when paired with proper waste segregation and innovative recycling techniques.
9.	Bichu et al., (2023) (23)	Environmental and economical	Aligner materials Manufacturing process	Innovations like shape-memory polymers, direct 3D-printed aligners, and bioactive materials are pivotal for improving the predictability and applications of CAT. Coupled with the adoption of biodegradable thermoplastics and increased environmental awareness among stakeholders, these advancements could align orthodontics with sustainability goals.
10.	Ahmed et al., (2023) (24)	Environmental and economical	Carbon footprint Recycling	CAT impacts the environment through travel, material use, energy consumption, and waste, with limited data on its footprint. Challenges include low awareness of carbon goals and resource constraints. Adopting sustainable practices like the four Rs, integrating environmental and economic considerations, and fostering education and research can align orthodontics help mitigate climate change
11.	Campobasso et al., (2023) (25)	Environmental and economical	Aligner materials cost	Evaluated the use of innovative 3D-printed materials, such as Polyamide-12 (PA-12) and Shape Memory Polymers (SMPs), in aligners. These advanced materials enable the creation of customized, metal-free, and fully digital aligners. With exceptional mechanical, aesthetic, and biocompatibility properties, PA-12 and SMPs hold significant potential for enhancing efficient, patient-specific aligner treatments.
12.	Caelli et al., (2023) (26)	Environmental	Aligner materials Manufacturing process	This study compares two methods for producing clear dental aligners: thermoforming and direct 3D printing using Additive Manufacturing (AM) with Digital Light Processing (DLP) technology. A Life Cycle Assessment (LCA) reveals that direct printing offers significant environmental benefits, including reduced raw material and electricity consumption, leading to lower environmental impacts and waste generation compared to thermoforming. This demonstrates that AM technology can contribute to more sustainable healthcare practices.
13.	Peter et al., (2022) (27)	Environmental and economical	Waste management Aligner cost Carbon emission Manufacturing process	The growing use of clear aligners in orthodontics has raised concerns over their environmental impact, particularly with increased treatment regimens and plastic waste. While regulations on single-use plastics are being enforced globally, orthodontic treatment should consider the 4R principle—reduce, reuse, recycle, and recover—particularly by focusing on reducing the frequency of aligner tray changes. Research into more sustainable materials and a take-back policy for used aligners is crucial to minimize waste and environmental burden.

### Major themes identified

Descriptive thematic synthesis of the 13 included studies identified five major themes related to the environmental sustainability of clear aligner therapy: (1) aligner materials and manufacturing processes, (2) waste management and recycling practices, (3) microplastics and environmental contamination, (4) carbon emissions and life-cycle environmental impacts, and (5) emerging sustainability strategies. Although the studies consistently recognized the environmental challenges associated with clear aligner therapy, differences were observed in the proposed mitigation strategies and the strength of the available evidence.

Theme 1: Aligner materials and manufacturing processes

Nine of the included studies examined the environmental implications of aligner materials and manufacturing technologies. Across these studies, conventional thermoplastic polymers such as PET-G, polypropylene, polycarbonate, and thermoplastic polyurethane were consistently identified as important contributors to plastic waste because of their petroleum-based origin and limited recyclability. Several studies proposed innovations including direct three-dimensional (3D) printing, shape-memory polymers, and alternative polymer formulations as potential approaches to reduce material consumption and manufacturing waste (17, 25, 27). Life-cycle assessment studies further demonstrated that direct 3D printing technologies, particularly Digital Light Processing (DLP), may reduce raw material consumption, electricity use, and manufacturing waste compared with conventional thermoforming techniques. However, despite promising preliminary findings, the included studies emphasized that additional environmental life-cycle assessments are required before these technologies can be considered sustainable alternatives in routine orthodontic practice (20, 26).

Theme 2: Waste management and recycling practices

Eight studies explored waste management and recycling practices associated with clear aligner therapy. There was broad agreement that discarded aligners represent an increasing source of plastic waste because they are generally designed for single-patient use and are replaced frequently throughout treatment. Most studies advocated implementation of the principles of Reduce, Reuse, Recycle, and Rethink (4Rs) together with improved waste segregation and manufacturer-supported take-back programs (18, 22, 27). Although recycling initiatives were consistently proposed as a strategy to reduce environmental impact, the studies also identified substantial barriers, including limited recycling infrastructure, contamination of used aligners, economic constraints, and the absence of closed-loop recycling systems capable of producing new aligners from recycled material. Consequently, existing recycling programs remain limited in scope and effectiveness.

Theme 3: Microplastics and environmental contamination

Six studies highlighted concerns regarding the generation of microplastics during the clinical use and disposal of clear aligners. Environmental contamination resulting from degradation of thermoplastic polymers was consistently identified as a potential concern, particularly following landfill disposal or incineration. While several studies also discussed possible human health implications of microplastic exposure, the available evidence primarily focused on environmental contamination rather than establishing direct clinical health effects associated with clear aligner therapy. The included studies emphasized the need for standardized methods to quantify microplastic release and to evaluate the long-term environmental consequences associated with aligner materials throughout their life cycle (13, 19, 20).

Theme 4: Carbon emissions and environmental impacts

Seven studies evaluated the broader environmental impacts associated with clear aligner therapy, including greenhouse gas emissions generated during material production, manufacturing, transportation, and disposal. Across the included literature, the environmental footprint of clear aligners was recognized as extending beyond plastic waste alone, encompassing energy consumption and resource utilization throughout the product life cycle. Several studies suggested that digital workflows, optimized manufacturing processes, shorter treatment protocols, and reduced material consumption may contribute to lowering carbon emissions. However, the evidence remains limited because few studies have performed comprehensive life-cycle assessments comparing different aligner manufacturing and disposal pathways (21, 27).

Theme 5: Evidence gaps and future research priorities

Across all themes, the included studies consistently identified a lack of standardized environmental assessment methods and high-quality life-cycle analyses for clear aligner therapy. Although numerous sustainability strategies including biodegradable materials, direct 3D printing, recycling initiatives, and circular economy approaches have been proposed, supporting evidence remains limited and is largely based on narrative reviews or laboratory investigations rather than comprehensive environmental assessments. This highlights the need for standardized reporting frameworks and robust comparative studies to better evaluate the environmental performance of current and emerging aligner technologies (20–27). (Table 3)

**Table 3:** Thematic synthesis of the environmental sustainability evidence in clear aligner therapy.

Theme	Number of studies	Main findings	Knowledge gaps
Materials and manufacturing	9	Conventional thermoplastics dominate; 3D printing and shape-memory polymers may reduce waste	Limited life-cycle assessment evidence
Waste management and recycling	8	Recycling and 4Rs widely recommended	Limited infrastructure and closed-loop recycling
Microplastics	6	Environmental contamination from polymer degradation is a concern	Lack of standardized measurement methods
Carbon emissions	7	Manufacturing and disposal contribute to greenhouse gas emissions	Few comparative carbon footprint studies
Sustainability strategies	8	Circular economy, digital workflows, material innovation	Limited real-world implementation and evaluation

## Discussion

This scoping review mapped the available evidence on the environmental impact of clear aligner therapy (CAT) and identified five major themes: aligner materials and manufacturing processes, waste management and recycling, microplastic contamination, carbon emissions, and emerging sustainability strategies. Overall, the available evidence indicates that the environmental burden of CAT extends beyond plastic waste and includes resource consumption, greenhouse gas emissions, and challenges associated with the disposal of thermoplastic polymers. Most of the included studies were narrative reviews or laboratory investigations, highlighting the limited availability of primary environmental assessment studies (11, 13, 17–27).

The findings are consistent with previous literature showing that commonly used aligner materials, including PET-G, polypropylene, polycarbonate, thermoplastic polyurethane, and ethylene-vinyl acetate, possess desirable mechanical and clinical properties but also contribute to environmental concerns because they are petroleum-based polymers with limited recycling potential (2, 7, 13, 23, 28). Although these materials generally demonstrate acceptable biocompatibility, prolonged intraoral use may result in material degradation and microplastic release, raising environmental concerns that extend beyond clinical performance (13, 28–31). Several studies identified innovative manufacturing approaches, including direct three-dimensional (3D) printing, shape-memory polymers, and biodegradable materials, as potential strategies to reduce material consumption and manufacturing waste (17, 20, 23, 25, 26, 32–35, 40–42). However, the long-term environmental advantages of these technologies remain uncertain because few studies have evaluated them using standardized life-cycle assessment (LCA) methodologies. Consequently, current evidence supports these technologies as promising alternatives rather than established sustainable solutions (25, 26, 42).

An important finding of this review is that sustainability in orthodontics should extend beyond recycling alone. While recycling initiatives and take-back programmes may reduce plastic waste, several studies emphasised broader circular economy principles, including reducing unnecessary material consumption, improving product design, optimizing manufacturing efficiency, and implementing extended producer responsibility programmes (17, 18, 22, 24, 27). These approaches are likely to provide greater long-term environmental benefits than relying solely on end-of-life recycling. Digital orthodontic workflows may also contribute to environmental sustainability. Direct 3D printing, digital treatment planning, and optimized manufacturing processes have been reported to reduce raw material consumption and production waste compared with conventional thermoforming techniques (23, 25, 26). Nevertheless, robust comparative environmental studies remain scarce, and further LCA-based investigations are needed before definitive conclusions regarding environmental superiority can be drawn.

Although several included studies discussed the potential biological effects of microplastics released from aligner materials, including oxidative stress and inflammatory responses (13, 19, 29, 36–39), the present review focused primarily on environmental sustainability rather than human health outcomes. Current evidence directly linking aligner-derived microplastics to adverse clinical health effects remains limited, and future research should clearly distinguish environmental contamination from patient-related health risks while evaluating the complete life cycle of aligner materials (13, 29, 36–39, 43, 44).

Overall, the available evidence suggests that improving material selection, adopting circular economy principles, optimizing manufacturing technologies, and strengthening waste management practices may reduce the environmental burden associated with clear aligner therapy. However, standardized environmental reporting and high-quality prospective environmental assessment studies remain essential to support evidence-based sustainable orthodontic practice.

### Future directions

The findings of this review demonstrate several important knowledge gaps. Future research should prioritize standardized life-cycle assessment (LCA) studies comparing conventional thermoforming and emerging manufacturing technologies, including direct 3D printing. Prospective environmental assessments evaluating carbon emissions, resource consumption, and waste generation throughout the complete life cycle of clear aligners are also required. Additional investigation into biodegradable polymers, alternative sustainable materials, circular economy models, and manufacturer-supported product stewardship programs will be essential to support environmentally responsible orthodontic practice. Furthermore, standardized reporting frameworks for environmental outcomes would improve comparability across future studies. Future research should also evaluate whether material modifications, including antimicrobial or nanoparticle-based surface technologies, can improve material durability and biocompatibility without increasing environmental impacts throughout the product life cycle (45, 46).

### Limitations

This review has several limitations. Only English-language publications from five electronic databases were included, which may have resulted in the omission of relevant studies published in other languages or indexed elsewhere. The evidence base was limited to 13 studies with heterogeneous study designs, most of which were narrative reviews or laboratory investigations rather than primary environmental assessment studies. As a scoping review, the objective was to map the available evidence rather than quantitatively synthesize findings; therefore, no meta-analysis was performed. Additionally, inter-reviewer agreement statistics were not calculated, although all disagreements during study selection were resolved through discussion and consensus.

## Conclusion

This scoping review demonstrates that clear aligner therapy presents important environmental challenges related to plastic waste generation, resource consumption, manufacturing processes, and greenhouse gas emissions. Available evidence suggests that innovations in aligner materials, optimized manufacturing technologies, circular waste management strategies, and improved recycling initiatives may reduce the environmental burden associated with clear aligner therapy. However, current evidence remains limited, with relatively few studies employing comprehensive environmental assessment methods such as life-cycle assessment. Further high-quality research is required to support evidence-based sustainable practices and guide future policy, manufacturing, and clinical decision-making aligned with Sustainable Development Goals 3, 9, 12, and 13.

## Data Availability

The original contributions presented in the study are included in the article/Supplementary Material, further inquiries can be directed to the corresponding author.
